# 847. Exploring the Benefits and Side Effects of Testosterone Administration in Major Burn Patients

**DOI:** 10.1093/jbcr/irag033.317

**Published:** 2026-04-06

**Authors:** Malak Al Wahaibi, Anthony Papp

**Affiliations:** Vancouver General Hospital BC Burn Unit, Vancouver, BC; Vancouver General Hospital BC Burn Unit, Vancouver, BC

## Abstract

**Introduction:**

Severe burn injuries trigger a hypermetabolic and catabolic response, leading to muscle wasting, and weight loss. Anabolic and agents like insulin, metformin, growth hormone, and Testosterone have been investigated to mitigate this response. Oxandrolone was widely adopted in burn care but has recently been withdrawn from multiple institutes, highlighting the need for alternatives. Intramuscular testosterone is used in our burn center as a practical therapy. The aim of this study was to evaluate its effects on weight stability, and to compare hospital course, mortality, and adverse effects between testosterone-treated and control patients with major burns.

**Methods:**

A single-center retrospective cohort study including patients admitted with >20% TBSA burns between January 2018 to July 2025. Patients were divided into an intervention group receiving testosterone, and a control group receiving standard care without testosterone. Exclusion criteria was incomplete or missing data, liver disease, death within two weeks of admission. Variables collected were demographics, weekly weights, comorbidities, TBSA, BAUX score, length of stay, mortality, thromboembolic events, and liver function tests. The primary objective was to compare weight trends between groups; secondary objectives were to compare hospital stay, mortality, and adverse effects.

**Results:**

A total of 82 patients met inclusion criteria (26 intervention, 56 control). Age, sex, and inhalation injury were comparable. Mean TBSA was higher in the intervention group (45.2% vs 31.4%). Mean hospital stay was longer in the testosterone group (98.3 vs 44.2 days, *p*<.0001), reflecting greater burn severity. Mortality rates were similar (15.4% vs 10.7%). Weight declined in both groups, but the control group had a steeper slope (–2.19 kg/week vs –0.29 kg/week). Although not statistically significant (*p*=.095), the trend suggested testosterone attenuated catabolic weight loss. Rates of LFT derangements, DVT, and PE were comparable between groups.

**Conclusions:**

Testosterone therapy in major burn patients was associated with relative weight stability compared with controls. Safety outcomes, including liver function abnormalities and thromboembolic events, were similar between groups. The observed trends suggest testosterone may attenuate catabolic weight loss. Testosterone represents a practical, accessible therapy with promise as an alternative after oxandrolone withdrawal.

**Applicability of Research to Practice:**

Testosterone offers a practical, accessible, and well-tolerated anabolic therapy in severe burns. By attenuating weight loss and preserving lean mass during the hypermetabolic phase, it has the potential to improve rehabilitation and long-term outcomes. These findings support its integration into clinical practice as a promising adjunctive therapy in burn care, pending validation in larger, multicenter studies.

**Funding for the study:**

N/A.

